# Prevalence and initiation of statin therapy in the oldest old—a longitudinal population-based study

**DOI:** 10.1007/s00228-022-03343-w

**Published:** 2022-07-05

**Authors:** Helena Sundvall, Sigurd Vitols, Susanna M. Wallerstedt, Johan Fastbom

**Affiliations:** 1 Department of Medicine, Clinical Pharmacology Unit, Karolinska Institutet Stockholm, Karolinska University Hospital, Z5:00, SE-171 76 Solna, Stockholm, Sweden; 2 Department of Pharmacology, Sahlgrenska Academy, University of Gothenburg, HTA-Centrum, Sahlgrenska University Hospital, Gothenburg, Sweden; 3 Aging Research Center, Department of Neurobiology, Care Sciences and Society, Karolinska Institutet and Stockholm University, Stockholm, Sweden

**Keywords:** Dose, Incidence, Older people, Pharmacoepidemiology, Prevalence, Register, Statins

## Abstract

**Purpose:**

To investigate the prevalence and initiation of statins as well as treatment intensity in the oldest old, with younger olds as a reference.

**Methods:**

A population-based cohort was used, including record-linked data from the Total Population Register, the Swedish Prescribed Drug Register, and the Swedish Patient Register. In each year over the study period (2009–2015), statin use was described in individuals 85 years or older and 65–84 years of age, and initiation rates were calculated among individuals with no statin treatment during a preceding 3-year period.

**Results:**

A total of 1,764,836 individuals ≥ 65 years in 2009, increasing to 2,022,764 in 2015, were included in the analyses. In individuals 85 years or older, the prevalence of statin therapy increased from 11% in 2009 to 16% in 2015, the corresponding initiation rates being 1.3% and 1.7%, respectively. Corresponding prevalence and incidence figures in 65–84-year-olds were 23 to 25% and 3.0 to 3.3%, respectively. Overall, the proportion of individuals initiating statin with high-intensity treatment (atorvastatin ≥ 40 mg or rosuvastatin ≥ 20 mg) in the oldest old increased from 1 to 36% during the study period, and a similar increase was seen in the younger age group. Over the study years, the presence of an established indication for statin treatment varied between 70 and 76% in the oldest old and between 30 and 39% in the younger olds.

**Conclusion:**

Prevalence and initiation of statin therapy are increasing among the oldest old, despite the fact that randomized controlled trials focusing on this age group are lacking and safety signals are difficult to detect.

**Supplementary Information:**

The online version contains supplementary material available at 10.1007/s00228-022-03343-w.

## Introduction

Statin treatment is important in cardiovascular disease and has been proven effective in reducing major vascular events and mortality, especially in secondary prevention [1–7]. However, the large randomized controlled studies with statins have not included a significant proportion of older individuals. For example, the pivotal 4S study only included individuals 35–70 years of age [7], and the primary prevention WOSCOPS study included only males aged 45–64 years [6]. The latest meta-analysis by the Cholesterol Treatment Trialists (CTT) collaboration, focusing on advancing age on major endpoints in 28 statin trials, showed that the relative risk reduction was less in participants over 75 years of age and not statistically significant among those without the previous vascular disease [8]. There are limitations also in this meta-analysis—among others a low proportion of older patients (8% were 75 years or older) and that the included trial populations were selected with fewer comorbidities than normally seen at older ages. Another recent meta-analysis, however, suggests beneficial effects in those 75 years or older [9]. Nevertheless, the relatively high use of statins among the oldest old could be questioned due to the short life expectancy and sparsity of evidence for an effect in this age group.

In a previous population-based study on statin use in older people according to age and indication [10], we found a relatively high prevalence of statin users. That is, 17% of all individuals ≥ 80 years of age were on statins in 2010, compared with 23% in the age group 65–80 years. Therefore, as randomized controlled trials of the benefit-risk balance for statin therapy in this age group are sparse, we further studied the use of statins in the oldest old, by investigating the prevalence and initiation of statins, as well as treatment intensity, in those 85 years or older, with younger olds as a reference. We also explored the use according to initiating specialty, with a focus on statin type, treatment intensity, and treatment duration.

## Method

### Study population

The study population included all individuals 65 years or older, who lived in Sweden during the period January 1, 2009, to December 31, 2015, or part of this period.

For each year, the study population was defined as follows: Individuals were included in the yearly population from the year they turned 65. For example, an individual born in 1945 was included in the population from 2010 onwards. Individuals who died during the study period were excluded from the population from the year after they died. Thus, an individual who died in 2012 was included in the 2012 population, but not from 2013 and onwards. Individuals who immigrated were included in the population from the year after immigration and individuals who emigrated were excluded from the population from the year of emigration. A total of 107 individuals with incomplete or ambiguous immigration/emigration dates, or re-used personal identity numbers, were excluded from the study.

### Data sources

Data were collected from three population-based registers, linked by the unique personal identity number:

- The Total Population Register at Statistics Sweden provided information about who were residents in Sweden, as well as dates of deaths and moving into/out of the country during the study period [11, 12].
- The Swedish Prescribed Drug Register (SPDR) at the Swedish National Board of Health and Welfare provided information on all prescribed drugs dispensed in any pharmacy in Sweden [13].
- The Swedish Patient Register [14] contains data from all inpatient stays and specialized outpatient visits in Sweden. The diagnoses are registered according to the International Statistical Classification of Diseases and Related Health Problems (ICD-10).

### Definitions and procedures

The prevalence of statin use was calculated each year from 2009 to 2015. An individual was classified as a prevalent statin user each year if he/she filled statin prescriptions (codes C10AA or C10BA in the Anatomical Therapeutic Chemical classification system (ATC) [15] covering at least 75% of the year. Swedish regulations stipulate drugs can be dispensed for a maximum of 3-month use at a time. Thus, our definition of a statin user was ≥ 3 filled statin prescriptions over a year. Individuals who had at least one drug dispensed within the multi-dose drug-dispensing (MDD) system and therefore had drugs delivered every fortnight were classified as statin users if they had ≥ 20 statin dispensations over the year.

For each year from 2009 to 2015, we also calculated the proportions of the different statin types used among the statin users. A specific statin (simvastatin, fluvastatin, pravastatin, atorvastatin, cerivastatin, rosuvastatin, or pitavastatin; ATC codes C10AA01, C10AA03-C10AA05, C10AA07, C10AA08) or combination drugs (simvastatin/ezetimibe, pravastatin/fenofibrate, simvastatin/fenofibrate, atorvastatin/ezetimibe, or rosuvastatin/ezetimibe; ATC codes C10BA02-C10BA06) was classified as used by each individual and year if he/she had at least one filled prescription, or one multi-dose dispensation, of the specific statin that year. The proportion of each statin was then calculated by dividing the number of individuals using the statin by the total number of statin users.

For each year, 2009 to 2015, a person was defined as a statin initiator that year if he/she filled a statin prescription (ATC codes C10AA or C10BA) for the first time during this year, not having used a statin in the previous 3 years. A few individuals, who initiated treatment a second time, within the study period, after a washout period of 3 years, were defined as statin initiators twice.

For each filled prescription, a start date and an end date were defined. The start date was defined as the date of fill. The end date was calculated by estimating the number of days the prescription would last and adding it to the date of fill. The number of days a prescription would last was calculated by dividing the amount of drug dispensed by the prescribed dosage, for ordinary prescriptions, and set to 14 days for drugs delivered within the MDD system. The procedure has been described previously [16].

Treatment duration was defined as the difference between the first date of the first filled prescription and the calculated end date of the last filled prescription. A gap of 6 months between the end date of one prescription and the first date of the next was allowed. When there was a gap of more than 6 months, the last day of treatment was defined as the end date of the last filled prescription before this gap. Sensitivity analyses were performed with an alternative that allowed a gap of 3 months. For those deceased during treatment, the death date was considered the end date of treatment. When calculating treatment duration, only individuals still alive during the follow-up period were included.

Statin daily dose intensity was defined according to guidelines by the American College of Cardiology/American Heart Association (ACC/AHA) [17], as low intensity: fluvastatin < 80 mg, pitavastatin < 2 mg, pravastatin < 40 mg, or simvastatin < 20 mg; moderate intensity: atorvastatin ≥ 10 < 40 mg, fluvastatin ≥ 80 mg, pitavastatin ≥ 2 mg, pravastatin ≥ 40 mg, rosuvastatin ≥ 10 < 20 mg, or simvastatin ≥ 20 mg; and high intensity: atorvastatin ≥ 40 mg or rosuvastatin ≥ 2 0 mg. The prescribed daily dose (PDD) was calculated by dividing the prescribed daily dose by the defined daily dose (DDD) according to the World Health Organization (WHO) [15], which was for rosuvastatin 10 mg, atorvastatin 20 mg, simvastatin, and pravastatin 30 mg, and fluvastatin 60 mg.

Information regarding the setting of the initiating prescriber was retrieved from the SPDR and merged into five categories: general practice, internal medicine (also including cardiology and neurology since these specialties are included in the department of internal medicine in many smaller hospitals in Sweden), surgery, geriatrics, and other, which included the remaining specialties.

ICD-10 diagnoses from inpatient and specialized outpatient care, recorded over the 5-year period preceding statin initiation, were retrieved from the Swedish Patient register. The following established indications were extracted, being considered relevant for statin treatment: ischemic heart disease (ICD-10 I20-I25), cerebrovascular disease (ICD-10 I63-I67, I69.3), TIA (ICD-10 G45), cerebral vascular syndromes (ICD-10 G46), atherosclerosis (ICD-10 I70), and diabetes (ICD-10 E10-14).

Descriptive analyses were performed using STATA (Version 14) and SPSS (IBM SPSS Statistics for Windows, version 23.0, Armonk, NY).

## Results

### Study population

**Fig. 1:**
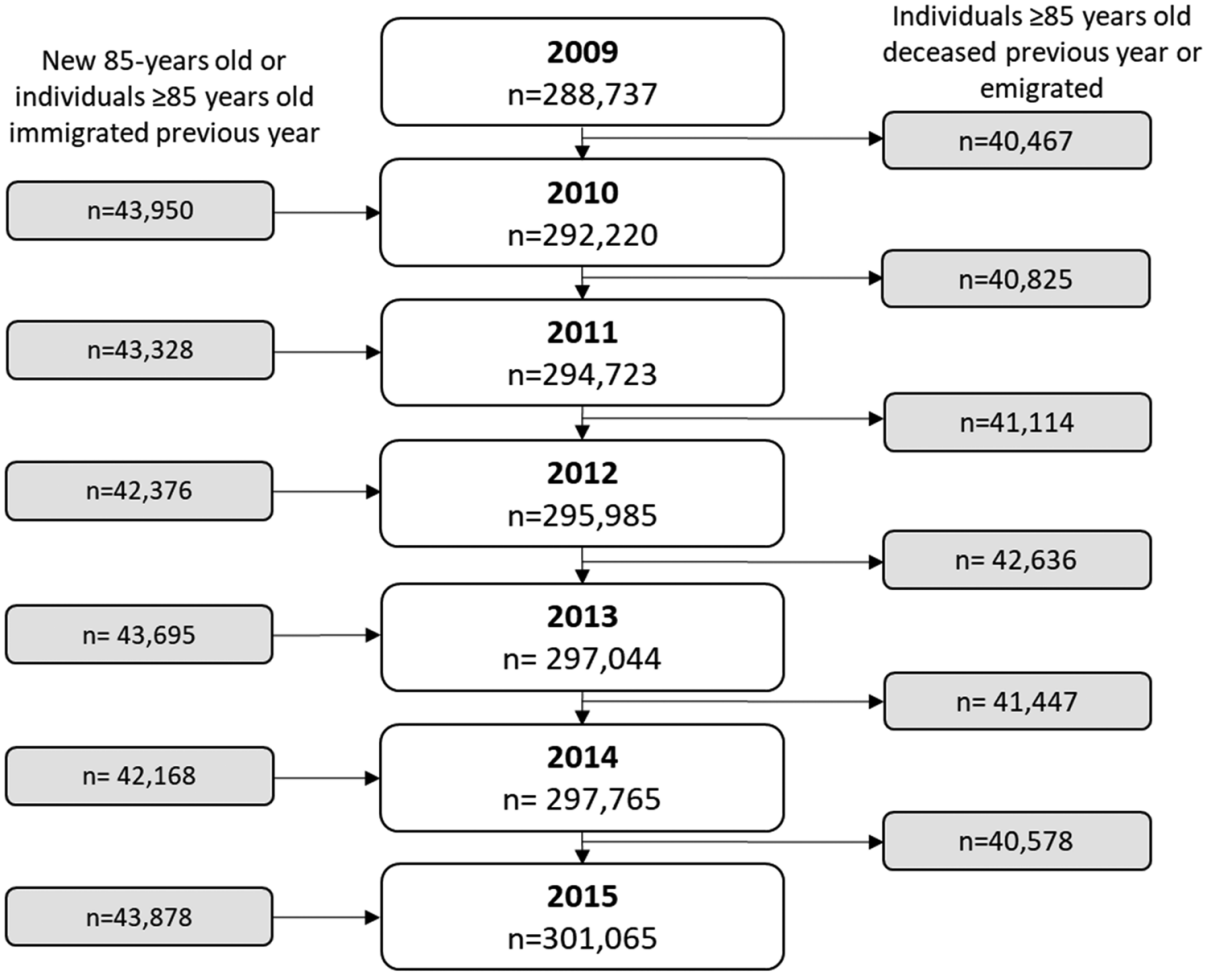
Flowchart of the study population 2009–2015

The population of Swedish inhabitants, 85 years or older, consisted of 288,737 individuals in 2009 and 301,065 individuals in 2015 (Fig. 1, Table 1). The proportion of women was 66% in 2009 and 65% in 2015 and the mean age was 88.9 in 2009 and 89.3 in 2015. The population of younger olds, 65–84 years, was 1,476,099 individuals in 2009 and 1,721,699 in 2015 (Appendix 1).

**Table 1:** Characteristics of older olds (≥ 85 years of age). Values are provided as the number of individuals (percentage) if not stated otherwise

		2009	2010	2011	2012	2013	2014	2015
**All (*n*)**		288,737	292,220	294,723	295,985	297,044	297,765	301,065
**Age, mean (SD)**		88.9 (3.5)	89.0 (3.5)	89.0 (3.5)	89.1 (3.5)	89.2 (3.5)	89.2 (3.6)	89.3 (3.6)
**Females**		191,155 (66.2)	192,978 (66.0)	194,011 (65.8)	194,308 (65.7)	194,255 (65.4)	194,029 (65.2)	195,213 (64.8)
**Statin prevalence**	All	31,402 (10.9)	35,948 (12.3)	38,275 (13.0)	40,827 (13.8)	43,104 (14.5)	45,422 (15.3)	49,399 (16.4)
	Men	13,877 (14.2)	15,916 (16.0)	17,155 (17.0)	18,649 (18.3)	19,868 (19.3)	21,133 (20.4)	23,093 (21.8)
	Women	17,520 (9.2)	20,032 (10.4)	21,120 (10.9)	22,178 (11.4)	23,236 (12.0)	24,289 (12.5)	26,306 (13.5)
**Statin type**^a^	Simvastatin	28,712 (87.1)	33,156 (90.8)	35,177 (90.8)	37,100 (89.4)	37,702 (85.7)	37,372 (80.4)	37,408 (73.8)
	Atorvastatin	2581 (7.8)	1866 (5.1)	2047 (5.3)	2746 (6.6)	4511 (10.3)	7267 (15.6)	11,172 (22.1)
	Pravastatin	1190 (3.6)	1109 (5.1)	1082 (2.8)	1079 (2.6)	1117 (2.5)	1068 (2.3)	1117 (2.2)
	Rosuvastatin	290 (0.9)	356 (1.0)	420 (1.1)	548 (1.3)	630 (1.4)	742 (1.6)	935 (1.8)
	Fluvastatin	174 (0.5)	45 (0.1)	31 (0.1)	21 (0.1)	16 (0.0)	14 (0.0)	13 (0.0)
	Simvastatin + ezetemibe	0 (0.0)	0 (0.0)	3 (0.0)	4 (0.0)	10 (0.0)	15 (0.0)	21 (0.0)

*SD* standard deviation

^a^One individual can contribute to ≥ 1 substance

### Statin prevalence

Among the oldest old (≥ 85 years), 11% were statin users in 2009 and 16% in 2015. The use of simvastatin decreased from 87% in 2009 to 74% in 2015 (*p* < 0.001) while atorvastatin increased from 8% in 2009 to 22% in 2015 (*p* < 0.001). Pravastatin and rosuvastatin were used to a smaller extent, as well as fluvastatin and the combination simvastatin/ezetimibe (Table 2). Among the younger old (65–84 years), 23% were statin users in 2009 and 25% in 2015 (Appendix 2).

**Table 2:** Prescribing details for statin initiators among the oldest old (≥ 85 years). Values are provided as number of individuals (percentage) if not stated otherwise

		2009	2010	2011	2012	2013	2014	2015
**Statin initiators**	All	3417 (1.3)	3536 (1.4)	3525 (1.4)	3720 (1.5)	3694 (1.5)	4045 (1.6)	4156 (1.7)
	Men	1347 (1.6)	1425 (1.7)	1469 (1.8)	1547 (1.9)	1562 (1.9)	1615 (2.0)	1706 (2.1)
	Women	2070 (1.2)	2111 (1.2)	2056 (1.2)	2173 (1.3)	2132 (1.2)	2430 (1.4)	2450 (1.5)
**Statin type**	Simvastatin	3347 (98.0)	3462 (97.9)	3411 (96.8)	3209 (86.3)	2274 (61.6)	1719 (42.5)	1281 (30.8)
	Atorvastatin	49 (1.4)	54 (1.5)	95 (2.7)	489 (13.1)	1,393 (37.7)	2,289 (56.6)	2,842 (68.4)
	Other	21 (0.6)	20 (0.6)	19 (0.5)	22 (0.6)	27 (0.7)	37 (0.9)	33 (0.8)
**PDD, mean (SD)**^a^	Simvastatin	1.02 (0.62)	1.05 (0.63)	1.05 (0.59)	1.02 (0.57)	1.04 (0.57)	1.01 (0.55)	1.02 (0.55)
	Atorvastatin	1.74 (1.35)	2.01 (1.21)	2.75 (1.95)	2.14 (1.41)	1.95 (1.20)	1.80 (1.12)	1.75 (1.09)
**Intensity**^b^	Low	471 (13.8)	439 (12.4)	383 (10.9)	342 (9.2)	171 (4.6)	159 (4.0)	109 (2.6)
	Moderate	2922 (85.5)	3068 (86.8)	3084 (87.5)	3088 (83.0)	2706 (73.3)	2635 (65.1)	2534 (61.0)
	High	24 (0.7)	29 (0.8)	58 (1.6)	290 (7.8)	817 (22.1)	1251 (30.9)	1513 (36.4)
**Presence of established indication**^c^	All	2401 (70.3)	2521 (71.3)	2672 (75.8)	2764 (74.3)	2724 (73.7)	2933 (72.5)	3124 (75.2)
	Men	993 (73.7)	1050 (73.7)	1153 (78.5)	1163 (75.2)	1173 (75.1)	1210 (74.9)	1330 (78.0)
	Women	1408 (68.0)	1471 (69.7)	1519 (73.9)	1601 (73.7)	1551 (72.7)	1723 (70.9)	1794 (73.2)
**Treatment duration**	≤ 100 days	804 (23.5)	830 (23.5)	835 (23.7)	788 (21.2)	885 (24.0)	953 (23.6)	N/A
	> 100 days to ≤ 12 months	784 (22.9)	837 (23.7)	858 (24.3)	750 (20.2)	856 (23.2)	810 (20.2)	N/A
	> 12 to ≤ 24 months	728 (21.3)	785 (22.2)	688 (19.5)	654 (17.6)	465 (12.6)	N/A	N/A
	> 24 months	1101 (32.2)	1084 (30.7)	1144 (32.5)	1528 (41.1)	1488 (40.3)	N/A	N/A

*N/A* not applicable, *SD* standard deviation

^a^Prescribed daily dose (PDD) was calculated by dividing the prescribed daily dose by the defined daily dose (DDD) according to WHO [16] for atorvastatin 20 mg and for simvastatin 30 mg

^b^Statin daily dose intensity was defined according to ACC/AHA guidelines [18], as low intensity: fluvastatin < 80 mg, pitavastatin < 2 mg, pravastatin < 40 mg, and simvastatin < 20 mg. Moderate intensity: atorvastatin ≥ 10 < 40 mg, fluvastatin ≥ 80 mg, pitavastatin ≥ 2 mg, pravastatin ≥ 40 mg, rosuvastatin ≥ 10 < 20 mg, or simvastatin ≥ 20 mg. High intensity: atorvastatin ≥ 40 mg or rosuvastatin ≥ 20 mg

^c^The following established indications relevant to statin treatment, were identified: ischemic heart disease (ICD-10 I20-I25), cerebrovascular disease (ICD-10 I63-I67, I69.3), TIA (ICD-10 G45), cerebral vascular syndromes (ICD-10 G46), atherosclerosis (ICD-10 I70), and diabetes (ICD-10 E10-14)

### Statin initiators

In the oldest age group (≥ 85 years), statin initiation increased during the study period, from 1.3 to 1.7% (*p* < 0.001) (Table 2). In 2009, simvastatin was the most frequently initiated statin, with 98% receiving this drug, compared with 31% in 2015. During the study period, atorvastatin became more common and was in 2014 and 2015 the most frequently initiated statin, with 57% of the new prescriptions in 2014 and 68% in 2015 (Table 2). Throughout the study period, initiation of a statin was more frequent in the younger age group (65–84 years), 3.0% in 2009 and 3.3% in 2015 (*p* < 0.001) (Appendix 2).

During the study period, a shift towards more intensive treatment was seen in the oldest old. In 2009, 1% was being prescribed high-intensity treatment, whereas the corresponding figure in 2015 was 36% (*p* < 0.001) (Table 2). A similar shift towards high-intensity treatment was seen in the younger olds. The mean PDD at statin initiation was higher for atorvastatin and varied more during the study period, compared with simvastatin.

For statin initiators in the oldest age group, the proportion who discontinued treatment within the first 100 days was 21 to 24% in 2009–2014 (Table 2). In all, 32% in 2009, and 40% in 2013, continued their statin treatment for more than 2 years. An established indication was found for 70% in 2009 and for 75% in 2015. The corresponding figures for the younger olds were 29% and 39%, respectively (Appendix 2).

### Statin prescribing by prescriber setting

**Fig. 2:**
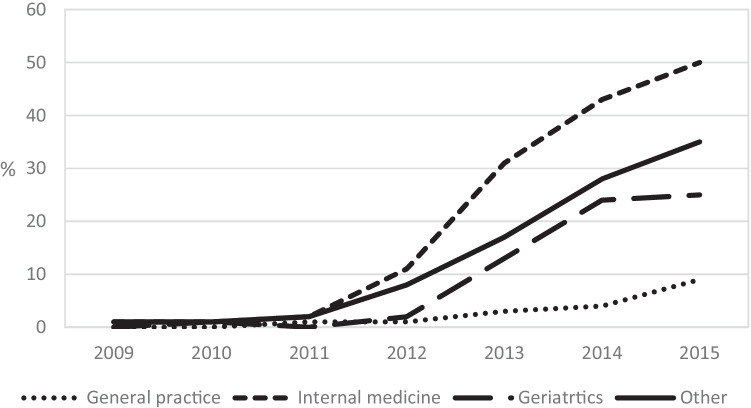
Proportion of high-intensity treatment, i.e., either atorvastatin ≥ 40 mg or rosuvastatin ≥ 20 mg, initiated over the study years (2009–2915), by the setting of the initiating prescriber

During the entire study period, statins in the oldest old (≥ 85 years) were most often initiated from internal medicine, 46% in 2009 and 53% in 2015; the second most frequent initiating setting being primary care, 27% in 2009 and 23% in 2015 (Table 3). Over the study period, PDD and treatment intensity were higher in those who had their statin treatment initiated from internal medicine compared with primary care, and the proportion of high-intensity treatment increased during the study period (Fig. 2). In younger olds (65–84 years), statins were most often initiated from primary care, 49% in 2009 and 52% in 2015 (Appendix 3).

**Table 3:** Prescribing details for the oldest old (≥ 85 years), according to initiating setting. Values are provided as number of individuals (percentage) if not stated otherwise

			2009	2010	2011	2012	2013	2014	2015
**General practice**			916 (26.8)	785 (22.2)	698 (19.8)	723 (19.4)	710 (19.2)	864 (21.4)	936 (22.5)
	Statin type	Simvastatin	891 (97.3)	773 (98.5)	673 (96.4)	659 (91.2)	528 (74.4)	537 (62.2)	456 (48.7)
		Atorvastatin	16 (1.7)	4 (0.5)	17 (2.4)	55 (7.6)	171 (24.1)	312 (36.1)	465 (49.7)
		Other	9 (1.0)	8 (1.0)	8 (1.2)	9 (1.2)	11 (1.5)	15 (1.7)	15 (1.6)
	PDD, mean (SD)^a^	Simvastatin	0.72 (0.39)	0.72 (0.39)	0.73 (0.33)	0.72 (0.31)	0.72 (0.31)	0.74 (0.32)	0.75 (0.33)
		Atorvastatin	0.81 (0.40)	0.75 (0.29)	1.39 (1.06)	1.15 (0.78)	1.00 (0.70)	0.97 (0.53)	1.05 (0.67)
	Established indication^b^	All	331 (36.1)	268 (34.1)	265 (38.0)	240 (33.2)	241 (33.9)	304 (35.2)	320 (34.2)
		Men	119 (40.2)	117 (42.5)	109 (44.0)	98 (35.4)	95 (38.3)	108 (37.8)	119 (37.9)
		Women	212 (34.2)	151 (29.6)	156 (34.7)	142 (31.8)	146 (31.6)	196 (33.9)	201 (32.3)
**Internal medicine**			1581 (46.3)	1854 (52.4)	1984 (56.3)	2115 (56.8)	2167 (58.7)	2305 (57.0)	2225 (53.5)
	Statin type	Simvastatin	1551 (98.1)	1807 (97.5)	1914 (96.5)	1763 (83.4)	1144 (52.8)	704 (30.5)	438 (19.7)
		Atorvastatin	23 (1.5)	38 (2.0)	64 (3.2)	342 (16.2)	1,012 (46.7)	1,586 (68.8)	1,774 (79.7)
		Other	7 (0.4)	9 (0.5)	6 (0.3)	10 (0.5)	11 (0.5)	15 (0.7)	13 (0.6)
	PDD, mean (SD)^a^	Simvastatin	1.17 (0.67)	1.17 (0.66)	1.17 (0.63)	1.13 (0.62)	1.16 (0.62)	1.15 (0.60)	1.21 (0.63)
		Atorvastatin	2.547 (1.37)	2.28 (1.22)	3.07 (1.99)	2.34 (1.48)	2.14 (1.21)	1.97 (1.15)	1.95 (1.12)
	Established indication^b^	All	1361 (86.1)	1581 (85.3)	1738 (87.6)	1833 (86.7)	1837 (84.8)	1935 (83.9)	1991 (89.5)
		Men	598 (85.9)	666 (85.4)	754 (87.6)	799 (86.4)	817 (84.5)	831 (85.1)	893 (89.9)
		Women	763 (86.2)	915 (85.2)	984 (87.6)	1034 (86.9)	1020 (85.0	1104 (83.3)	1098 (89.1)
**Geriatrics**			132 (3.9)	176 (5.0)	169 (4.8)	185 (5.0)	175 (4.7)	172 (4.3)	193 (4.6)
	Statin type	Simvastatin	130 (98.5)	174 (98.9)	167 (98.8)	178 (96.2)	142 (81.1)	117 (68.0)	79 (40.9)
		Atorvastatin	2 (1.5)	2 (1.1)	2 (1.2)	7 (3.8)	32 (18.3)	54 (31.4)	114 (59.1)
		Other	0 (0.0)	0 (0.0)	0 (0.0)	0 (0.0)	1 (0.6)	1 (0.6)	0 (0.0)
	PDD, mean (SD)^a^	Simvastatin	0.85 (0.38)	0.85 (0.40)	0.91 (0.44)	0.85 (0.36)	0.99 (0.35)	0.98 (0.46)	0.94 (0.40)
		Atorvastatin	N/A	N/A	N/A	1.21 (076)	1.47 (0.83)	1.49 (0.72)	1.53 (0.93)
	Established indication^b^	All	212 (91.7)	161 (91.5)	151 (89.3)	172 (93.0)	156 (89.1)	157 (91.3)	182 (94.3)
		Men	51 (94.4)	60 (93.7)	64 (90.1)	70 (89.7)	61 (87.1)	57 (95.0)	64 (92.8)
		Women	70 (89.7)	101 (90.2)	87 (88.8)	102 (95.3)	5 (90.5)	100 (89.3)	118 (95.2)
**Other**			788 (23.1)	721 (20.4)	674 (19.1)	697 (18.7)	642 (17.4)	704 (17.4)	802 (19.3)
	Statin type	Simvastatin	775 (98.3)	708 (98.2)	657 (97.5)	609 (87.4)	460 (71.7)	361 (51.3)	308 (38.4)
		Atorvastatin	8 (1.0)	10 (1.4)	12 (1.8)	85 (12.2)	178 (27.7)	337 (47.9)	489 (61.0)
		Other	5 (0.6)	3 (0.4)	5 (0.7)	3 (0.4)	4 (0.6)	6 (0.8)	5 (0.6)
	PDD, mean (SD)^a^	Simvastatin	1.10 (0.62)	1.14 (0.67)	1.07 (0.54)	1.08 (0.57)	1.16 (0.57)	1.16 (0.57)	1.14 (0.54)
		Atorvastatin	1.83 (1.44)	1.51 (1.05)	3.28 (1.99)	2.07 (1.18)	1.92 (1.11)	1.81 (1.09)	1.75 (1.04)
	Established indication^b^	All	588 (74.6)	511 (70.9)	518 (76.9)	519 (74.5)	490 (76.3)	537 (76.3)	631 (78.7)
		Men	225 (74.7)	207 (67.6)	226 (78.2)	196 (73.4)	200 (72.2)	214 (73.3)	254 (77.0)
		Women	363 (74.5)	304 (73.3)	292 (75.8)	323 (75.1)	290 (79.5)	323 (78.4)	377 (79.9)

*SD* standard deviation

^a^Prescribed daily dose (PDD) was calculated by dividing the prescribed daily dose by the defined daily dose (DDD) according to WHO [16] for atorvastatin 20 mg and for simvastatin 30 mg

^b^The following established indications relevant to statin treatment were identified: ischemic heart disease (ICD-10 I20-I25), cerebrovascular disease (ICD-10 I63-I67, I69.3), TIA (ICD-10 G45), cerebral vascular syndromes (ICD-10 G46), atherosclerosis (ICD-10 I70), and diabetes (ICD-10 E10-14)

## Discussion

In this nationwide population-based study, we found that the overall prevalence of statin users in the oldest old increased from 11% in 2009 to 16% in 2015. In this age group, more individuals initiated statin treatment at the end of the study period, and a shift towards high-intensity treatment was seen. In comparison, statin use was more common in younger olds over the entire study period but did not increase as conspicuously. A similar shift towards high-intensity statin treatment was, however, noted.

The overall increase in statin prevalence in older people is similar to that reported in other studies [18]. However, our finding that statin use increased markedly also in the oldest old deserves attention. Although a recent systematic review and meta-analysis suggest beneficial effects in those 75 years or older [9], the benefit-risk balance in the oldest old in general, including frail individuals with short life expectancy, is still uncertain. Perhaps an ongoing RCT, including 18,000 patients 70 years or older and studying atorvastatin 40 mg, may add further insights (NCT02099123); the size of that study may perhaps allow subgroup analyses of the oldest old and those who develop frailty over the study period. Nevertheless, it must be acknowledged that studying benefits and risks in the oldest old imply challenges; heterogeneity and gradually declining health may aggravate the potential to detect efficacy and safety aspects of preventive treatment.

An increase in statin prevalence among the oldest old could be explained by an expanded prescribing of statins at younger ages, continuing over the years, or by an increased initiation of treatment at older ages. Our results suggest that both alternatives may have contributed to the increased statin prevalence in this age group. Indeed, clinicians may find it difficult to withdraw statin treatment as patients age; there is no clearcut point at the individual level where such treatment does not add any benefit. Furthermore, our results show an increased initiation of statin treatment over the years.

In the study population as a whole, nearly every other statin initiator in 2015 received their first prescription from primary care. This finding is consistent with a previous study [19]. In the oldest olds, however, more than every second statin initiator instead had their first prescription from an internal medicine setting. Geriatricians, on the other hand, initiate statin treatment only to a small extent, a finding that may be explained by the fact that they treat the oldest and most frail patients with a short life expectancy and therefore uncertainties regarding beneficial effects. Furthermore, almost nine in ten of these patients had an underlying established indication for statin treatment. These results are in line with our previous study, where old age was positively associated with the presence of an established indication, suggesting that this age group is more often prescribed statins for secondary prevention [10].

During the study period, a shift towards higher intensity treatment was seen in statin initiators. In 2015, as much as 36% of oldest olds were prescribed high-intensity treatment, compared with only 1% in the oldest age group in 2009. The same shift was seen in the younger age group. This change can be explained by the fact that simvastatin was replaced by atorvastatin as the most prescribed statin during these years, which, in turn, may be due to the fact that the patent for atorvastatin expired in 2012, resulting in a marked price reduction making simvastatin and atorvastatin similarly priced. Another explanation could be a trend towards choosing higher intensity treatment with statins, especially in the hospital setting. ACC/AHA guidelines recommend high-intensity statin treatment for secondary prevention in men and women < 75 years of age and a large observational study found a small but significant survival advantage associated with high intensity, compared with moderate-intensity, statins [20]. We found that, in 2015, eight in ten individuals in the oldest old group received atorvastatin when initiated from internal medicine, whereas simvastatin and atorvastatin were about equally prescribed upon statin initiation in primary care. We have not found many similar studies analyzing the dose of atorvastatin compared with simvastatin, but one earlier study reported that PDD, in relation to DDD, was in general higher for atorvastatin [21].

### Strengths and limitations

An important strength of this study is that it comprises the entire older population in Sweden. Additionally, it covers a long period of time, from 2006 to 2015, which allows examination of longer time trends in the initiation of drug therapy. Another strength is that the wealth of information in the SPDR makes it possible to study not only drug use but also doses. Limitations include that the SPDR only records prescription drugs dispensed at pharmacies. Drugs administered in hospitals or from drug storerooms in nursing homes are not recorded in the register, potentially leading to an underestimation of the actual drug exposure. Furthermore, a general limitation of studies based on drug register data is that dispensed drugs may not reflect what is actually taken by the patient. On the other hand, repeated drug fills indicate that the patient is actually using the drug over time. Another limitation is that the information on diagnoses in the present study was based only on data recorded from inpatient and specialized outpatient care. However, the vast majority of established indications consist of conditions that require hospital care, and therefore, this limitation may have had less effects on the results. Furthermore, it may be regarded as a limitation that we did not differentiate our results between secondary prevention (ischemic heart disease and/or cerebrovascular disease) and primary prevention. Indeed, statin treatment for secondary prevention is reportedly less common in those 80 years or older compared with younger olds [10].

## Conclusion

This register-based study shows that statin prevalence and incidence, as well as treatment intensity and duration in statin initiators, is increasing in the oldest old. As many older people are exposed to statins—drugs that may also imply a risk of adverse reactions—and also quite often at high doses, further studies evaluating the benefit-risk balance, including the potential quality-of-life gain/loss, are needed to guide clinicians in the process of prescribing.

## Supplementary Information

Below is the link to the electronic supplementary material.

Supplementary file1 (DOCX 28 KB)

Supplementary file2 (DOCX 30 KB)

Supplementary file3 (DOCX 37 KB)

## Data Availability

Dr Fastbom and Mrs Sundvall had full access to all data and take responsibility for the integrity of the data and accuracy of the data analysis.

## Code availability

Dr Fastbom has full access to the data registry (non-identifiable person data). Acquisition, analysis, and analysis of data: Sundvall, Fastbom.
