# Supplementary material for: Prevalence and initiation of statin therapy in the oldest old—a longitudinal population-based study
Source: Eur J Clin Pharmacol. 2022 Jul 5;78(9):1459–67. doi: 10.1007/s00228-022-03343-w (PMC9365718; doi:10.1007/s00228-022-03343-w)
Supplement: Supplementary file 1 — Supplementary file1 (DOCX 28 KB) [file 228_2022_3343_MOESM1_ESM.docx]

Appendix 1 Characteristics of younger olds (65-84 years of age). Values are provided as number of individuals (percentage) if not stated otherwise.

|  |  | **2009** | **2010** | **2011** | **2012** | **2013** | **2014** | **2015** |
| --- | --- | --- | --- | --- | --- | --- | --- | --- |
| **All (n)** |  | 1,476,099 | 1,519,120 | 1,563,927 | 1,608,363 | 1,649,460 | 1,688,567 | 1,721,699 |
| **Mean age (SD)** |  | 73.0 (5.7) | 72.8 (5.7) | 72.7 (5.7) | 72.7 (5.6) | 72.6 (5.6) | 72.7 (5.5) | 72.7 (5.4) |
| **Females (%)** |  | 53.2 | 53.0 | 52.7 | 52.5 | 52.4 | 52.2 | 52.1 |
| **Statin prevalence, n (%)** | All | 339,590 (23.0) | 360,003 (23.7) | 363,082 (23.2) | 376,762 (23.4) | 393,557 (23.9) | 407,721 (24.1) | 425,365 (24.7) |
|  | Men | 177,216 (25.7) | 190,087 (26.6) | 194,348 (26.3) | 204,295 (26.8) | 215,519 (27.4) | 225,428 (27.9) | 236,601 (28.7) |
|  | Women | 162,374 (20.7) | 169,916 (21.1) | 168,734 (20.5) | 172,467 (20.4) | 178,038 (20.6) | 182,293 (20.7) | 188,764 (21.1) |
| **Statin type, %** ^a^ | Simvastatin | 294,369 (80.7) | 311,472 (83.3) | 309,049 (82.0) | 312,745 (79.4) | 305,453 (73.9) | 289,580 (67.7) | 271,236 (60.7) |
|  | Atorvastatin | 46,418 (12.7) | 39,413 (10.5) | 43,475 (11.5) | 55,255 (14.0) | 80,983 (19.6) | 110,221 (25.8) | 145,653 (32.6) |
|  | Pravastatin | 11,664 (3.2) | 10,166 (2.7) | 9,662 (2.6) | 9,316 (2.4) | 8,714 (2.1) | 8,085 (1.9) | 7,697 (1.7) |
|  | Rosuvastatin | 10,288 (2.8) | 12,337 (3.3) | 14,322 (3.8) | 16,109 (4.1) | 17,570 (4.3) | 19,168 (4.5) | 21,703 (4.9) |
|  | Fluvastatin | 1,813 (0.5) | 329 (0.1) | 204 (0.1) | 155 (0.0) | 122 (0.0) | 113 (0.0) | 103 (0.0) |
|  | Simvastatin+ezetemibe | 0 (0.0) | 0 (0.0) | 135 (0.0) | 374 (0.1) | 488 (0.1) | 467 (0.1) | 493 (0.1) |

SD = standard deviation

^a^ One individual can contribute to ≥1 substance
