# Supplementary material for: Prevalence and initiation of statin therapy in the oldest old—a longitudinal population-based study
Source: Eur J Clin Pharmacol. 2022 Jul 5;78(9):1459–67. doi: 10.1007/s00228-022-03343-w (PMC9365718; doi:10.1007/s00228-022-03343-w)
Supplement: Supplementary file 2 — Supplementary file2 (DOCX 30 KB) [file 228_2022_3343_MOESM2_ESM.docx]

Appendix 2. Prescribing details for statin initiators among the younger olds (65-84 years). Values are provided as number of individuals (percentage) if not stated otherwise.

|  |  | **2009** | **2010** | **2011** | **2012** | **2013** | **2014** | **2015** |
| --- | --- | --- | --- | --- | --- | --- | --- | --- |
| **Statin initiators** | All | 34,498 (3.0) | 38,047 (3.3) | 36,470 (3.0) | 37,659 (3.1) | 37,781 (3.0) | 40,528 (3.2) | 43,268 (3.3) |
|  | Men | 15,995 (3.1) | 18,386 (3.5) | 18,301 (3.4) | 19,313 (3.5) | 18,845 (3.3) | 20,740 (3.6) | 21,920 (3.7) |
|  | Women | 18,503 (3.0) | 19,661 (3.1) | 18,169 (2.8) | 18,346 (2.8) | 18,936 (2.8) | 19,788 (2.8) | 21,348 (3.0) |
| **Statin type** | Simvastatin | 33,380 (96.8) | 36,598 (96.2) | 34,435 (94.4) | 31,311 (83.1) | 23,514 (62.2) | 18,702 (46.1) | 14,207 (32.8) |
|  | Atorvastatin | 698 (2.0) | 946 (2.5) | 1,466 (4.0) | 5,688 (15.1) | 13,681 (36.2) | 21,153 (52.2) | 28,214 (65.2) |
|  | Other | 420 (1.2) | 503 (1.3) | 569 (1.6) | 660 (1.8) | 586 (1.6) | 673 (1.7) | 847 (2.0) |
| **PDD, mean (SD)** ^a^ | Simvastatin | 0.95 (0.56) | 0.98 (0.57) | 1.01 (0.57) | 0.99 (0.54) | 0.95 (0.52) | 0.93 (0.50) | 0.92 (0.51) |
|  | Atorvastatin | 1.76 (1.39) | 2.09 (1.63) | 2.34 (1.69) | 2.17 (1.48) | 1.90 (1.31) | 1.82 (1.25) | 1.73 (1.20) |
| **Intensity** ^b^ | Low | 4,074 (11.8) | 4,054 (10-7) | 3,446 (9.5) | 3,040 (8.1) | 2,352 (6.2) | 1,961 (4.8) | 1,574 (3.6) |
|  | Moderate | 30,145 (87.4) | 33,536 (88.1) | 32,175 (88.2) | 31,266 (83.0) | 28,254 (74.8) | 27,941 (68.9) | 28,422 (65.7) |
|  | High | 279 (0.81) | 457 (1.2) | 849 (2.3) | 3,353 (8.9) | 7,175 (19.0) | 10,626 (26.2) | 13,272 (30.7) |
| **Presence of established indication** ^c^ | All | 12,715 (36.9) | 14,454 (38.0) | 14,380 (39.4) | 14,828 (39.4) | 14,858 (39.3) | 15,746 (38.9) | 16,931 (39.1) |
|  | Men | 6,829 (42.7) | 7,952 (43.3) | 7,992 (43.7) | 8,515 (44.1) | 8,324 (44.2) | 8,930 (43.1) | 9,692 (44.2) |
|  | Women | 5,886 (31.8) | 6,502 (33.1) | 6,388 (35.2) | 6,313 (34.5) | 6,534 (34.5) | 6,816 (34.5) | 7,239 (33.9) |
| **Treatment duration** | ≤100 days | 5,742 (16.6) | 6,478 (17.0) | 7,121 (19.5) | 6,344 (16.8) | 6,898 (18.3) | 7,230 (17.8) | N/A |
|  | >100 days to ≤12 months | 5,393 (15.6) | 6,322 (16.6) | 5,494 (15.1) | 5,065 (13.5) | 5,359 (14.2) | 5,328 (13.2) | N/A |
|  | >12 to ≤ 24 months | 5,206 (15.1) | 5,547 (14.6) | 4,513 (12.4) | 4,255 (11.3) | 3,884 (10.3) | 27,970 (69.0) | N/A |
|  | >24 months | 18,157 (52.6) | 19,700 (51.8) | 19,342 (53.0) | 21,995 (58.4) | 21,640 (57.3) | N/A | N/A |

N/A = not applicable, SD = standard deviation

^a^ Prescribed daily dose (PDD) was calculated by dividing the prescribed daily dose by the Defined Daily Dose (DDD) according to WHO [16] for atorvastatin 20 mg and for simvastatin 30 mg.
^b^ Statin daily dose intensity was defined according to ACC/AHA guidelines [18], as low intensity: fluvastatin <80 mg, pitavastatin <2 mg, pravastatin <40 mg and simvastatin <20 mg. Moderate intensity: atorvastatin ≥10 <40 mg, fluvastatin ≥80 mg, pitavastatin ≥2 mg, pravastatin ≥40 mg, rosuvastatin ≥10 <20 mg or simvastatin ≥20 mg. High intensity: atorvastatin ≥40 mg or rosuvastatin ≥20 mg.
^c^ The following established indications relevant to statin treatment, were identified: ischemic heart disease (ICD-10 I20-I25), cerebrovascular disease (ICD-10 I63-I67, I69.3), TIA (ICD-10 G45), cerebral vascular syndromes (ICD-10 G46), atherosclerosis (ICD-10 I70), and diabetes (ICD-10 E10-14).
