# Supplementary material for: Prevalence and initiation of statin therapy in the oldest old—a longitudinal population-based study
Source: Eur J Clin Pharmacol. 2022 Jul 5;78(9):1459–67. doi: 10.1007/s00228-022-03343-w (PMC9365718; doi:10.1007/s00228-022-03343-w)
Supplement: Supplementary file 3 — Supplementary file3 (DOCX 37 KB) [file 228_2022_3343_MOESM3_ESM.docx]

Appendix 3. Prescribing details for the younger old (65-84 years), according to initiating setting. Values are provided as number of individuals (percentage) if not stated otherwise.

|  |  |  | **2009** | **2010** | **2011** | **2012** | **2013** | **2014** | **2015** |
| --- | --- | --- | --- | --- | --- | --- | --- | --- | --- |
| **General practice** |  |  | 16,916 (49.0) | 18,426 (48.4) | 16,959 (46.5) | 17,573 (46.7) | 18,425 (48.8) | 20,227 (49.9) | 22,438 (51.9) |
|  | Statin type | Simvastatin | 16,451 (97.3) | 17,880 (97.0) | 16,292 (96.1) | 15,733 (89.5) | 13,577 (73.7) | 12,032 (59.5) | 9,682 (43.1) |
|  |  | Atorvastatin | 261 (1.5) | 287 (1.6) | 367 (2.2) | 1,536 (8.7) | 4,569 (24.8) | 7,841 (38.8) | 12,335 (55.0) |
|  |  | Other | 204 (1.2) | 259 (1.4) | 300 (1.8) | 304 (1.7) | 279 (1.5) | 354 (1.7) | 421 (1.9) |
|  | PDD, mean (SD) ^a^ | Simvastatin | 0.73 (0.35) | 0.73 (0.34) | 0.74 (0.32) | 0.74 (0.33) | 0.74 (0.33) | 0.76 (0.33) | 0.77 (0.35) |
|  |  | Atorvastatin | 1.09 (0.79) | 1.06 (0.51) | 1.14 (0.65) | 1.06 (0.58) | 1.01 (0.61) | 1.02 (0.60) | 1.04 (0.61) |
|  | Intensity ^b^ | Low | 2,814 (16.6) | 2,910 (15.8) | 2,505 (14.8) | 2,268 (12.9) | 1,955 (10.6) | 1,652 (8.2) | 1,313 (5.9) |
|  |  | Moderate | 14,057 (83.1) | 15,475 (84.0) | 14,376 (84.8) | 15,088 (85.9) | 15,929 (86.5) | 17,497 (86.5) | 19,226 (85.7) |
|  |  | High | 45 (0.3) | 41 (0.2) | 78 (0.5) | 217 (1.2) | 541 (2.9) | 1,078 (5.3) | 1,899 (8.5) |
|  | Established indication ^c^ | All | 2,384 (14.1) | 2,298 (12.5) | 2,149 (12.7) | 2,195 (12.5) | 2,373 (12.9) | 2,668 (13.2) | 2,782 (12.4) |
|  |  | Men | 1,208 (17.6) | 1,151 (14.9) | 1,079 (14.5) | 1,143 (14.7) | 1,182 (14.8) | 1,394 (15.2) | 1,497 (14.8) |
|  |  | Women | 1,176 (11.7) | 1,147 (10.7) | 1,070 (11.2) | 1,052 (10.7) | 1,191 (11.4) | 1,274 (11.5) | 1,285 (10.4) |
| **Internal medicine** |  |  | 10,688 (31.0) | 13,118 (34.5) | 13,311 (36.5) | 14,043 (37.3) | 13,774 (36.5) | 14,766 (36.4) | 14,629 (33.8) |
|  | Statin type | Simvastatin | 10,246 (95.9) | 12,467 (95.0) | 12,274 (92.2) | 10,627 (75.7) | 6,323 (45.9) | 3,962 (26.8) | 2,193 (15.0) |
|  |  | Atorvastatin | 321 (3.0) | 506 (3.9) | 891 (6.7) | 3,215 (22.9) | 7,273 (52.8) | 10,615 (71.9) | 12,158 (83.1) |
|  |  | Other | 121 (1.1) | 145 (1.1) | 146 (1.1) | 201 (1.4) | 178 (1.3) | 189 (1.3) | 278 (1.9) |
|  | PDD, mean (SD) ^a^ | Simvastatin | 1.28 (0.65) | 1.29 (0.65) | 1.31 (0.64) | 1.28 (0.60) | 1.26 (0.62) | 1.27 (0.62) | 1.31 (0.69) |
|  |  | Atorvastatin | 2.37 (1.56) | 2.72 (1.80) | 2.95 (1.74) | 2.76 (1.50) | 2.44 (1.33) | 2.37 (1.30) | 2.33 (1.29) |
|  | Intensity ^b^ | Low | 462 (4.3) | 535 (4.1) | 449 (3.4) | 390 (2.8) | 176 (1.3) | 128 (0.9) | 106 (0.7) |
|  |  | Moderate | 10,039 (93.9) | 12,240 (93.3) | 12,197 (91.6) | 11,112 (79.1) | 8,102 (58.8) | 6,777 (45.9) | 5,557 (38.0) |
|  |  | High | 187 (1.8) | 343 (2.6) | 665 (5.0) | 2,541 (18.1) | 5,496 (39.9) | 7,861 (53.2) | 8,966 (61.3) |
|  | Established indication ^c^ | All | 7,261 (67.9) | 9,068 (69.1) | 9,220 (69.3) | 9,657 (68.8) | 9,553 (69.4) | 10,125 (68.6) | 10,829 (74.0) |
|  |  | Men | 3,991 (69.9) | 5,123 (70.6) | 5,250 (70.0) | 5,733 (69.6) | 5,541 (709) | 5,942 (69.4) | 6,290 (75.7) |
|  |  | Women | 3,270 (65.6) | 3,945 (67.4) | 3,970 (68.3) | 3,924 (67.6) | 4,012 (67.4) | 4,183 (67.4) | 4,539 (71.8) |
| **Geriatrics** |  |  | 440 (1.3) | 522 (1.4) | 527 (1.4) | 553 (1.5) | 544 (1.4) | 587 (1.5) | 486 (1.1) |
|  | Statin type | Simvastatin | 433 98.4) | 509 (97.5) | 0 (96.8) | 489 (88.4) | 381 (70.0) | 252 (42.9) | 141 (29.0) |
|  |  | Atorvastatin | 4 (0.9) | 10 (1.9) | 11 (2.1) | 62 (11.2) | 160 (29.4) | 333 (56.7) | 344 (70.8) |
|  |  | Other | 3 (0.7) | 3 (0.6) | 6 (1.1) | 2 (0.4) | 3 (0.6) | 2 (0.3) | 1 (0.2) |
|  | PDD, mean (SD) ^a^ | Simvastatin | 0.93 (0.52) | 0.92 (0.40) | 1.01 (0.56) | 1.01 (0.41) | 1.00 (0.36) | 1.05 (0.53) | 1.03 (0.44) |
|  |  | Atorvastatin | 0.88 (0.25) | 3.00 (1.33) | 1.80 (1.22) | 1.51 (0.85) | 1.51 (0.98) | 1.75 (0.88) | 1.78 (0.87) |
|  | Intensity ^b^ | Low | 46 (10.5) | 49 (9.4) | 31 (5.9) | 24 (4.3) | 18 (3.3) | 12 (2.0) | 4 (0.8) |
|  |  | Moderate | 394 (89.5) | 465 (89.1) | 491 (93.2) | 503 (91.0) | 471 (86.6) | 369 (62.9) | 264 (54.3) |
|  |  | High | 0 | 8 (1.5) | 5 (0.9) | 26 (4.7) | 55 (10.1) | 206 (35.1) | 218 (44.9) |
|  | Established indication ^c^ | All | 355 (80.7) | 431 (82.6) | 436 (82.7) | 459 (83.0) | 449 (82.5) | 485 (82.6) | 445 (91.6) |
|  |  | Men | 173 (82.8) | 206 (84.4) | 211 (83.7) | 226 (82.5) | 212 (81.5) | 243 (82.1) | 224 (92.9) |
|  |  | Women | 182 (78.8) | 225 (80.9) | 225 (81.8) | 233 (82.5) | 237 (83.5) | 242 (83.2) | 221 (90.2) |
| **Other** |  |  | 6,454 (18.7) | 5,981 (15.7) | 5,673 (15.6) | 5,490 (14.6) | 5,03 (13.3) | 4,948 (12.2) | 5,715 (13.2) |
|  | Statin type | Simvastatin | 6,250 (96.8) | 5,742 (96.0) | 5,359 (94.5) | 4,462 (81.3) | 3,233 (64.2) | 2,456 (49.6) | 2,191 (38.3) |
|  |  | Atorvastatin | 112 (1.7) | 143 (2.4) | 197 (3.5) | 875 (15.9) | 1,679 (33.3) | 2,364 (47.8) | 3,377 (59.1) |
|  |  | Other | 92 (1.4) | 96 (1.6) | 117 (2.1) | 153 (2.8) | 126 (2.5) | 128 (2.6) | 147 (2.6) |
|  | PDD, mean (SD) ^a^ | Simvastatin | 1.02 (0.57) | 1.09 (0.60) | 1.14 (0.61) | 1.15 (0.58) | 1.93 (0.59) | 1.18 (0.59) | 1.19 (0.59) |
|  |  | Atorvastatin | 1.63 (1.23) | 1.85 (1.34) | 1.81 (1.42) | 2.00 (1.33) | 2.05 (1.28) | 2.00 (1.26) | 2.05 (1.22) |
|  | Intensity ^b^ | Low | 752 (11.7) | 560 (9.4) | 461 (8.1) | 358 (6.5) | 203 (4.0) | 169 (3.4) | 151 (2.6) |
|  |  | Moderate | 5,655 (87.6) | 5,356 (89.5) | 5,111 (90.1) | 4,563 (83.1) | 3,752 (74.5) | 3,298 (66.7) | 3,375 (59.1) |
|  |  | High | 47 (0.7) | 65 (1.1) | 101 (1.8) | 569 (10.4) | 1,083 (21.5) | 1,481 (29.9) | 2,189 (38.3) |
|  | Established indication ^c^ | All | 2,715 (42.1) | 2,657 (44.4) | 2,575 (45.4) | 2,517 (45.8) | 2,483 (49.3) | 2,468 (49.9) | 2,875 (50.3) |
|  |  | Men | 1,457 (45.6) | 1,472 (46.5) | 1,452 (46.5) | 1,413 (46.4) | 1,389 (49.6) | 1,351 (49.6) | 1,681 (51.7) |
|  |  | Women | 1,258 (38.6) | 1,185 (42.1) | 1,123 (44.0) | 1,104 (45.2) | 1,094 (48.8) | 1,117 (50.2) | 1,194 (48.4) |

SD = standard deviation

^a^ Prescribed daily dose (PDD) was calculated by dividing the prescribed daily dose by the Defined Daily Dose (DDD) according to WHO [16] for atorvastatin 20 mg and for simvastatin 30 mg.
^b^ Statin daily dose intensity was defined according to ACC/AHA guidelines [18], as low intensity: fluvastatin <80 mg, pitavastatin <2 mg, pravastatin <40 mg and simvastatin <20 mg. Moderate intensity: atorvastatin ≥10 <40 mg, fluvastatin ≥80 mg, pitavastatin ≥2 mg, pravastatin ≥40 mg, rosuvastatin ≥10 <20 mg or simvastatin ≥20 mg. High intensity: atorvastatin ≥40 mg or rosuvastatin ≥20 mg.
^c^ The following established indications relevant to statin treatment, were identified: ischemic heart disease (ICD-10 I20-I25), cerebrovascular disease (ICD-10 I63-I67, I69.3), TIA (ICD-10 G45), cerebral vascular syndromes (ICD-10 G46), atherosclerosis (ICD-10 I70), and diabetes (ICD-10 E10-14).
